# Training for hearing care providers

**DOI:** 10.2471/BLT.18.224659

**Published:** 2019-08-20

**Authors:** Mahmood F Bhutta, Xingkuan Bu, Patricia Castellanos de Muñoz, Suneela Garg, Kelvin Kong

**Affiliations:** a Department of Ear, Nose and Throat, Royal Sussex County Hospital, Eastern Road, Brighton BN2 5BE, England.; b Jiangsu Ear and Hearing Centre, Jiangsu Province Hospital, Nanjing, China.; c Centro de Audición CEDAF, Guatemala City, Guatemala.; d Department of Community Medicine, Maulana Azad Medical College, New Delhi, India.; e School of Medicine and Public Health, University of Newcastle, Callaghan, Australia.

## Abstract

The lack of an appropriately trained global hearing-care workforce is recognized as a barrier to developing and implementing services to treat ear and hearing disorders. In this article we examine some of the published literature on the current global workforce for ear and hearing care. We outline the status of both the primary-care workforce, including community health workers, and specialist services, including audiologists, ear, nose and throat specialists, speech and language therapists, and teachers of the deaf. We discuss models of training health workers in ear and hearing care, including the role of task-sharing and the challenges of training in low and middle-income countries. We structure the article by the components of ear and hearing care that may be delivered in isolation or in integrated models of care: primary care assessment and intervention; screening; hearing tests; hearing rehabilitation; middle-ear surgery; deaf services; and cochlear implant programmes. We highlight important knowledge gaps and areas for future research and reporting.

## Résumé

Le manque de prestataires de soins auditifs adéquatement formés à l'échelle mondiale est considéré comme un obstacle au développement et à la mise en œuvre de services destinés à traiter les troubles de l'oreille et de l'audition. Dans cet article, nous examinons des documents publiés au sujet de la main-d'œuvre mondiale actuelle au service des soins de l'oreille et de l'audition. Nous présentons l'état de la main-d'œuvre au service des soins primaires, et notamment des agents de santé communautaires, ainsi que l'état des services de spécialistes, et notamment des audiologistes, des spécialistes ORL, des thérapeutes de la parole et du langage et des enseignants pour les personnes sourdes. Nous étudions des modèles de formation des agents de santé axés sur les soins de l'oreille et de l'audition, et en particulier sur le rôle du partage des tâches et les problèmes liés à la formation dans les pays à revenu faible et intermédiaire. Cet article s'articule autour des différents aspects des soins de l'oreille et de l'audition, qui peuvent être fournis isolément ou dans le cadre de modèles intégrés de soins: évaluation des soins primaires et intervention; dépistage; examens auditifs; réhabilitation auditive; chirurgie de l'oreille moyenne; services pour les personnes sourdes; et programmes d'implantation cochléaire. Nous attirons l'attention sur d'importantes lacunes et sur les domaines sur lesquels pourraient porter les recherches et les rapports à l'avenir.

## Resumen

Se reconoce que la falta de trabajadores especializados en el cuidado de la salud auditiva a nivel mundial constituye un obstáculo para el desarrollo y la implementación de servicios de tratamiento de los trastornos auditivos y del oído. En este artículo examinamos parte de la literatura publicada sobre los trabajadores que actualmente se dedican al cuidado de la salud auditiva y del oído en todo el mundo. Describimos la situación de los trabajadores de atención primaria, incluidos los trabajadores sanitarios de la comunidad, y de los servicios especializados, incluidos los audiólogos, los especialistas en oído, nariz y garganta, los terapeutas del habla y del lenguaje, y los profesores de las personas sordas. Discutimos los modelos de formación de los trabajadores sanitarios en el cuidado de la salud auditiva y del oído y de la, incluyendo la función de la asignación de tareas y los retos de la formación en los países de ingresos bajos y medios. Estructuramos el artículo por los componentes del cuidado de la salud auditiva y del oído que se pueden prestar de forma aislada o en modelos integrados de atención: evaluación e intervención de la atención primaria; exámenes; pruebas de audición; rehabilitación de la audición; cirugía de oído medio; servicios para las personas sordas; y programas de implantes cocleares. Destacamos importantes lagunas de conocimiento y áreas para la investigación y presentación de informes en el futuro.

## ملخص

من المعروف أن النقص في القوى العاملة العالمية المدربة تدريباً ملائماً على رعاية السمع، يمثل عقبة في سبيل تطوير وتنفيذ الخدمات لعلاج اضطرابات الأذن والسمع.نقوم في هذه المقالة بالنظر في بعض الأدبيات المنشورة حول القوى العاملة العالمية الحالية لرعاية الأذن والسمع.نحن نوضح حالة كل من القوى العاملة للرعاية الأولية، بما في ذلك العاملين في القطاع الصحي المجتمعي، والخدمات المتخصصة، بما في ذلك أخصائيي السمع، وأخصائيي الأذن والأنف والحنجرة، وأخصائيي علاج النطق واللغة، ومعلمي الصم.نحن نناقش نماذج تدريب العاملين في القطاع الصحي في رعاية الأذن والسمع، بما في ذلك دور مشاركة المهام وتحديات التدريب في البلدان ذات الدخل المنخفض والدخل المتوسط. قمنا بوضع المقالة طبقاً لمكونات رعاية الأذن والسمع التي يمكن تقديمها بمفردها أو في نماذج متكاملة للرعاية: تقييم الرعاية الأولية والتدخل؛ والفحص؛ واختبارات السمع؛ وإعادة تأهيل السمع؛ وجراحة الأذن الوسطى؛ وخدمات الصم؛ وبرامج زراعة القوقعة.نحن نركز على الفجوات المعرفية الهامة، ومجالات البحث والتقارير المستقبلية.

## 摘要

开展和实施耳部和听力障碍治疗服务的一大阻碍是经适当培训的全球听力保健人员的匮乏。在本文中，我们考察了一些着重于当前全球耳部和听力保健人员的已发表文献。我们概述了初级保健人员（包括社区卫生工作者）和专业服务人员（包括听觉病矫治专家、耳鼻喉科专家、语言治疗师以及聋人教师）的状况。我们讨论了培训耳部和听力保健工作者的模式，包括任务分担的作用以及在中低收入国家进行培训所面临的挑战。本文根据耳部和听力保健的各个方面（以单独或综合护理的方式予以提供）：初级保健评估和干预；筛查；听力测试；听力康复；中耳手术；针对失聪人士的服务；以及人工耳蜗植入计划。我们着重阐释了未来研究和报告的重要知识鸿沟和相关领域。

## Резюме

Нехватка во всем мире надлежащим образом обученных специалистов по охране здоровья уха и слуха рассматривается как барьер для разработки и внедрения услуг по лечению уха и нарушений слуха. В данной статье проведено изучение опубликованной литературы, посвященной текущему состоянию трудовых ресурсов по охране здоровья уха и слуха во всем мире. Описано состояние как трудовых ресурсов первичного звена, включая общинных медицинских работников, так и специализированных услуг, включая аудиологов, оториноларингологов, логопедов, преподавателей для лиц с потерей слуха. Обсуждаются модели обучения специалистов здравоохранения в области охраны здоровья уха и слуха, включая роль распределения задач и проблемы обучения в странах с низким и средним уровнем дохода. Статья структурирована по компонентам охраны здоровья уха и слуха, которые можно обеспечить изолированно или в интегрированных моделях: первичная оценка и вмешательство, скрининговое обследование, испытания слуха, реабилитация при потере слуха, хирургия среднего уха, услуги для лиц с потерей слуха, программы установки кохлеарных имплантов. Акцентировано внимание на важных пробелах в знаниях, а также областях дальнейших исследований и отчетов.

## Introduction

The lack of an appropriately trained global hearing-care workforce is recognized as a barrier to developing and implementing services to treat ear and hearing disorders.^1^^–^^3^ This barrier is a particular issue in many low and middle-income countries, where a historical lack of awareness of the impact of such disorders, and a lack of prioritization against competing health needs, has led to low investment in relevant specialist resources.^2^^,^^3^

**Fig. 1:**
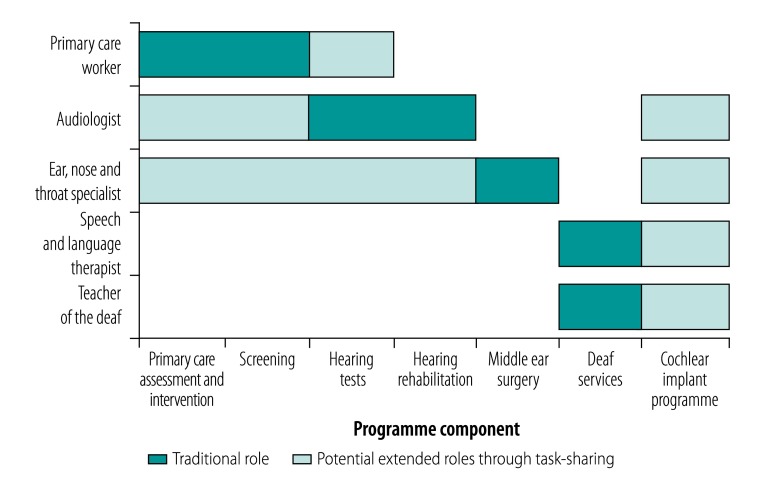
Potential human resources to deliver different components of an ear and hearing care programme

Both general (primary care) and specialized health workers can be used to deliver ear and hearing services (Fig. 1). They can be deployed in a variety of service delivery models, which may be informed by a needs assessment and evaluation of existing regional health infrastructure.^1^

The primary-care workforce for ear and hearing health may include community health workers, primary care nurses or primary care physicians, any of whom who may screen for disease and provide preventive or medical care. In most low-resource settings, there are inadequate numbers of doctors to provide primary hearing care, and in several countries nurses or community health workers fill this workforce gap. Community health workers have been defined as those who work predominantly in the community rather than in a health facility and have received some formal training in the tasks they perform, but do not have a certificate or degree-level education.^2^ In 2014 there were some estimated 5 million community health workers worldwide, with a particularly large workforce in India and Indonesia.^2^

Secondary or specialist care is traditionally delivered by audiologists who can test hearing and provide hearing aids; ear, nose and throat specialists who may offer medical or surgical treatment (surgery particularly for chronic suppurative ear disease); speech and language therapists who may assist adults and children with disabling hearing loss; and teachers of the deaf who can provide educational support to children with severe hearing loss.

**Fig. 2:**
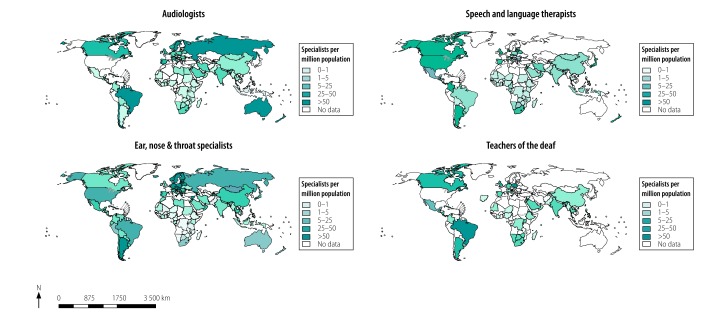
Global workforce of specialists in ear and hearing care

Notes: We plotted data collated from several workforce surveys,^3^^–^^10^ including raw data obtained for the World Health Organization survey^11^ and for the study by Verkerk et al.^12^ Such surveys rely on self-reporting and accuracy of data is therefore not assured. Where data were available from more than one source, we used the most recent figures.

Surveys conducted in the last few years reveal that in many low- and middle-income countries specialist workers in ear and hearing care are either sparse or non-existent (Fig. 2).^3–9,11,12^ In addition the tasks undertaken by such specialists may encompass a variety and variable complexity of roles. In the United Kingdom of Great Britain and Northern Ireland, for example, training pathways exist for audiometrists (who perform basic diagnostic hearing tests), hearing aid dispensers (who supply and fit hearing aids), health-care science practitioners (who provide a range of diagnostic tests and treatment) and clinical scientists (who provide tests and treatments that includes complex cases). In other countries, the names and roles of the workforce in audiology are similarly disparate.^3^ Among ear, nose and throat specialists, not all will operate on the ear; in the United Kingdom, for example, only 15% identify themselves as ear specialists,^13^ and surveys showed that facilities for complex ear surgery were poor or non-existent five out of 15 ear, nose and throat centres in Central and Eastern European countries,^12^ four out of six ear, nose and throat centres in Central American countries,^5^ and five out of 22 ear, nose and throat centres in Africa.^6^

Two parallel strategies can be pursued to fill the workforce gap in ear and hearing care. The first strategy is local capacity development, through the training and development of additional workers in the field of ear and hearing health. Even though locally delivered training may be preferred,^14^ delivering this training may be challenging: if there are inadequate human or physical resources in a country to deliver ear and hearing services, then usually these resources are likely to be inadequate to train or enable others to do the same. In the initial stages of workforce development, training may require collaboration with experts from outside of the region or country. The second strategy is task-sharing, which is the redistribution of tasks among different cadres of health workers, typically from specialist workers to those with less training. Such an approach requires a re-evaluation of traditional job roles, and an open yet critical analysis of where and how task-sharing may be both possible and appropriate.

In this article we assess opportunities for developing human resources in ear and hearing health. We present some of the relevant literature on training and development, and ask what has been shown to be effective or ineffective, and where there are opportunities and knowledge gaps. We identified relevant articles through a review of articles indexed in the PubMed® online database, using search terms “Training AND Audiology OR Otology OR Teacher Deaf OR Speech Language Therapy” as well as a search of the grey literature. We also look at examples of task-sharing and assess relevant outcomes reported from such initiatives. We structure the article by seven components of ear and hearing care that may be delivered in isolation or in integrated models of care: (i) primary-care assessment and intervention; (ii) screening; (iii) hearing tests; (iv) hearing rehabilitation; (v) middle-ear surgery; (vi) deaf services; and (vii) cochlear implant programmes (Fig. 1).

## Primary-care assessment and intervention

With an appropriately trained workforce, assessment and intervention for some ear and hearing disorders can potentially be undertaken in primary care. There are descriptions of programmes to train community health workers in such skills, and some anecdotes of success.^1^ However, we found that the current literature lacks evidence of important outcome of such efforts, especially changes to patient care or service delivery.

The World Health Organization’s (WHO’s) *Primary ear and hearing care training resource,*^15^ which is currently being revised, provides theoretical knowledge on ear and hearing disorders. The four training manuals include protocols for practical skills in examination of the ear (otoscopy); dry mopping of ear discharge; syringing of the ear; and assessment of hearing in babies and adults. These manuals have been used in several countries to train community health workers, including Brazil, Burkina Faso, China, Colombia, Fiji, Kenya, Malawi and Nigeria.^16^ Other regional or national resources for training community health workers are available, for example the *Aboriginal and Torres Strait Islander ear health manual*^17^from Australia, and the *Chinese ear and hearing care training manual.*^18^ Training programmes to date have reported learner outcomes of community health workers in terms of short-term improvements in knowledge,^16^ rather than ability to independently assess or manage ear and hearing disorders.

Other studies suggest that community health workers can acquire sufficient practical skills to assess and manage patients.^16^ Community health workers in India were able to perform a whispered voice test to screen for hearing loss in adults,^19^ and in Malawi they could identify patients with potential hearing problems.^20^ Several studies have shown that community health workers are able to capture images on digital otoscopy.^16^ In all these initiatives, however, the community health workers sent captured data for expert assessment and did not personally instigate diagnosis, management or intervention.

Further research on methods and outcomes of training is needed to provide evidence on if, how and to what extent training of community health workers enables them to undertake independent assessment and management of patients with ear and hearing disorders. Consequently, evidence will also show to what extent task-sharing may be possible in hearing care.

Achieving this higher level of ability in community health workers may be ambitious. It will likely require support from experts, both in delivering the training and in providing supervision in the initial stages of independent practice. In nations where such experts are available within the country, current technology may enable remote education and supervision.^1^ Where in-country expertise is lacking, partnerships with other countries may be helpful, notwithstanding cultural and language barriers. It is also unclear if the development of such expertise should be targeted at generic community health workers (who may have competing obligations, for example in delivering maternal or child health programmes) or whether training of a subset of specialized community hearing health workers would be preferable.

## Screening

Screening for hearing loss may include mass population screening for neonatal hearing loss or targeted screening of people classified as high-risk based on their geographical location or patient-specific factors (for example indigenous groups at risk of middle-ear disease or children who have suffered meningitis at risk of sensorineural hearing loss).

Automated devices have simplified several screening protocols. In the majority of European or other high-income settings, screening for neonatal hearing loss is undertaken by a nurse (or sometimes an audiologist) using automated otoacoustic emission or auditory brainstem response devices.^21^ Studies from India^22^ and Nigeria^23^ show that, after two weeks of training, community health workers can also perform automated neonatal screening using such devices. Screening in neonates by community health workers using a low-cost rattle made from wood and metal spheres has also been described in India,^24^ and in children and adults using a semi-automated mobile phone application (following only a few hours of training) in South Africa.^25^ Hence, the evidence suggests that automated screening of hearing is a feasible task for a variety of health workers, following only a relatively short period of training.

## Hearing rehabilitation

In many high-income countries, audiologists provide most of expertise in hearing assessment and rehabilitation, but task-sharing in this field has been described. A literature review found several studies reporting that community health workers were able to perform pure tone audiometry, the basic test of adult hearing, but details of the training given or the accuracy of the results were lacking in such studies.^16^ A study from the Dominican Republic reported that a charity developed a 3-month training programme for staff with at least secondary school education to perform basic hearing tests.^26^ Again, detailed methods or outcomes were not reported.

The fitting of hearing aids is also open to task-sharing. A survey of 62 countries globally in 2008 found that in 12 countries ear, nose and throat specialists undertook hearing tests, and in 11 countries they fitted hearing aids.^3^ In India, a cadre of science graduates were trained over six weeks to perform pure tone audiometry and to fit and maintain hearing aids for people with moderate to severe hearing loss, with successful long-term benefit to communication reported by the majority of users.^27^ Future technological advances may further simplify these tasks.

Provision of more complex audiology services requires a specialized workforce and longer training. In Bangladesh, a trial of accelerated training of community health workers over two weeks to carry out play audiometry (a method to test hearing where young children are asked to respond to sound by performing a task) proved unsuccessful.^28^ In the past, many audiologists visited neighbouring countries to obtain training, such as those from the Central and South America visiting Argentina, Mexico or the United States of America.^4^ Many nations now report in-country training programmes.^11^ Again, we found no published literature detailing the nature and duration of these training initiatives or learner outcomes.

Further studies are needed in this field, particularly those reporting methods and outcomes from the training of non-specialist workers to perform basic tests of hearing and to provide hearing rehabilitation services.

## Middle-ear surgery

The two operations for treating suppurative ear disease are tympanoplasty and mastoidectomy, both of which involve complex microsurgery on the temporal bone of the skull. Training in ear surgery is difficult, even in resource-rich environments. Standardized educational programmes exist in countries such as Australia, Canada, the United Kingdom and the United States, which typically comprise 5–6 years of specialist training in ear, nose and throat surgery,^29^ including training on cadaveric material.^30^ A survey in the United States showed an average of 4.5 years before trainees felt able to perform tympanoplasty or mastoidectomy independently.^31^ Data from the United Kingdom suggested that trainees in the last two years of training may still have suboptimal outcomes from tympanoplasty^32^ and be unable to perform mastoidectomy independently.^33^ One should note that such trainees are learning all aspects of ear, nose and throat surgery, not only otological surgery.

Targeted training in ear surgery may be available in low- and middle-income countries, but is often not possible due to a lack of in-country expertise, relevant equipment or (due to logistic or sometimes religious constraints) cadaveric material for rehearsal. Alternative simulation using plastic bones or virtual tools is possible, although inferior for learning outcomes and not without cost.^34^ Ear, nose and throat specialists may go abroad to train. For example, specialists from Malawi^35^ and Zimbabwe^7^ have been trained in South Africa, and those from Bhutan and Nepal in Malaysia,^8^ although outcomes from such training have not been reported. However, training in a foreign country may be difficult, because limited opportunities exist, medical qualifications may not be recognized internationally and the financial or language barriers can be substantial.

An alternative is to rely on visiting specialists to deliver training. In many low- and middle-income countries, specialists from high-resource settings visit on trips (missions) to provide a surgical service to the local patient population, and many will try to incorporate training into such trips. However, a recent survey from the United States of ear, nose and throat surgeons involved in global health found that the majority focused on missions, but that only 35% (125 out of 362) had been to the same hospital more than once, and that 93% (187 out of 202) went for no more than two weeks.^36^ Critics question the ability to provide meaningful training and local capacity development though such short-term and inconsistent visits. Interviews with ear, nose and throat trainees in Cambodia revealed they deemed these types of missions to be of little educational value.^37^

Coordinated missions can, however, be fruitful. A collaboration between ear, nose and throat departments in three North American medical schools (University of British Columbia, New York University School of Medicine and University of Ottawa) delivered in-country training in Uganda through frequent missions and a structured training programme.^38^ The project started in 2001 and the first independent mastoid operation by a Ugandan surgeon was performed four years later.

Another model is to provide prolonged in-country training by a visiting resident specialist. In Cambodia, two local ear, nose and throat trainees were trained by visiting ear, nose and throat specialists from the United Kingdom.^37^The first visiting surgeon was a continuous resident for six months and taught tympanoplasty. The second surgeon was a continuous resident for four months and taught mastoidectomy. At the end of this period, trainees were able to perform both operations independently, with high self-reported confidence and surgical success (for tympancoplasty: 88%; 100/113; tympanic membrane closure and 81%; 76/89; with improved hearing).^37^

It is uncertain if those with lower levels of background training could also perform ear surgery. Clinical or medical officers, who are not doctors, have been trained to deliver surgical care, such as laparotomy or caesarean section, in many countries,^39^ In some countries, such as Cameroon, Kenya, Malawi, Mali and Togo,^6^ this training includes performing simple ear, nose and throat operations, such as removal of foreign bodies, adenoidectomy or tonsillectomy. There are no reports of the outcomes from such training, and to date, the only record of trying to train such workers to perform tympanoplasty was said to be unsatisfactory.^40^

The existing literature suggests that the acquisition of skills in ear surgery is achieved via a focused, coordinated and consistent mentorship approach, taking place over several months or years. Future programmes for training in ear surgery should look to incorporate such ideals.

## Deaf services

Deaf services typically involve teachers of the deaf, and speech and language therapists. There are few published data on the methods or effectiveness of training for teachers of the deaf,^41^ making it difficult to know what a successful training programme might include.

Many countries have reported the existence of a training programme for speech and language therapists. In several countries in South America, and in some areas of India, audiology, and speech and language therapists are one profession, with a subspecialization.^42^^,^^43^ In some instances speech and language therapists have trained abroad. For example, therapists in Paraguay have trained in Brazil, therapists in Bolivia and Venezuela in the United States,^42^ and therapists in the Philippines and Singapore in Australia, the United Kingdom and the United States.^43^ Other examples document programmes in speech and language therapy that have been established with foreign assistance: for example in Sri Lanka and Uganda with volunteers from the United Kingdom,^44^^,^^45^ in Togo with volunteers from France,^46^ and in Viet Nam with volunteers from Australia.^47^ The institutions in Togo and Uganda have subsequently become regional centres in Africa, training speech and language therapists specialists in Benin, Gabon and Mali,^46^ and in Rwanda and the United Republic of Tanzania, respectively.^44^ Some authors have expressed concerns that foreign assistance in the development of training curricula for speech and language therapists risks marginalization of populations who may differ linguistically or culturally. For example this has occurred with the under-provision of speech and language therapists services to the black, economically disadvantaged population of South Africa.^48^

While the guiding principles of speech and language therapy or deaf education are universal, the specific nature of the training will be determined by the local culture and language (whether spoken or signed). Such differences means that sharing of training resources or learning across linguistic regions or borders may be difficult or even inappropriate. Outside assistance may be helpful to guide the set-up of services, but such initiatives need to remain sensitive to local needs.

## Cochlear implant programmes

Cochlear implants are surgically implanted devices for those affected by severe hearing loss. Implant programmes should ideally incorporate comprehensive pre-implant assessment and post-implant rehabilitation and support, which requires appropriately trained audiologists, ear, nose and throat surgeons, and teachers of the deaf or speech and language therapists. Anecdotally, cochlear implants have been performed in many low- and middle-income countries without such a comprehensive team, leading to concerns that such programmes may not be optimal. In general, data is lacking on outcomes from cochlear implantation in low- and middle-income countries, although data from several countries in South America and Asia show that in most locations implant use rates are over 95%.^49^^,^^50^

## Additional challenges

Other issues compound the challenges to human resources and training outlined above. One is the lack of specialist equipment, such as audiometers for performing hearing tests;^4^ hearing aids for rehabilitation; or microscope, drill and micro-instruments for performing ear surgery.^1^ Another is the retention of staff after training. In low- and middle-income countries, health workers in the charitable or public sector are often poorly remunerated, and so trained staff may leave to join the private sector. In many Eastern European countries, for example, otological surgery is more available in the private sector compared with the public sector.^12^ Processes to allow parallel public and private practice by health workers may be one mechanism to counteract this issue.^1^

## Conclusion

Training, development and task-sharing are strategies that can be used to counter the significant human resource gap for ear and hearing health. However, the existing literature inadequately addresses or documents the potential for these strategies.

In terms of training, most existing studies with community health workers have reported outcomes in terms of knowledge acquisition. We have not found any studies that evaluated whether community health workers may be able to independently assess and manage ear and hearing disorders in primary care. In the more complex tasks of undertaking hearing tests, hearing rehabilitation, middle-ear surgery and provision of deaf services, only a handful of studies describe training or task-sharing and of these few report outcomes.

Future studies should therefore report not only models of training, but also short and long-term outcomes of training, including the effects on delivery of care. Such reporting will inform those trying to emulate or translate such training to other contexts, and so help to optimize workforce development in ear and hearing health.
